# The Torque of Rotary F-ATPase Can Unfold Subunit Gamma If Rotor and Stator Are Cross-Linked

**DOI:** 10.1371/journal.pone.0053754

**Published:** 2013-01-03

**Authors:** Florian Hilbers, Wolfgang Junge, Hendrik Sielaff

**Affiliations:** 1 Department of Molecular Membrane Biology, MPI of Biophysics, Frankfurt, Germany; 2 Division of Biophysics, University of Osnabrück, Osnabrück, Germany; 3 Single-Molecule Microscopy Group, Jena University Hospital, Jena, Germany; Institute of Molecular Genetics IMG-CNR, Italy

## Abstract

During ATP hydrolysis by F_1_-ATPase subunit γ rotates in a hydrophobic bearing, formed by the N-terminal ends of the stator subunits (αβ)_3_. If the penultimate residue at the α-helical C-terminal end of subunit γ is artificially cross-linked (via an engineered disulfide bridge) with the bearing, the rotary function of F_1_ persists. This observation has been tentatively interpreted by the unfolding of the α-helix and swiveling rotation in some dihedral angles between lower residues. Here, we screened the domain between rotor and bearing where an artificial disulfide bridge did not impair the rotary ATPase activity. We newly engineered three mutants with double cysteines farther away from the C-terminus of subunit γ, while the results of three further mutants were published before. We found ATPase and rotary activity for mutants with cross-links in the single α-helical, C-terminal portion of subunit γ (from γ285 to γ276 in *E. coli*), and virtually no activity when the cross-link was placed farther down, where the C-terminal α-helix meets its N-terminal counterpart to form a supposedly stable coiled coil. In conclusion, only the C-terminal singular α-helix is prone to unwinding and can form a swivel joint, whereas the coiled coil portion seems to resist the enzyme's torque.

## Introduction

F-ATP synthase (F_O_F_1_) consists of two elastically coupled nanomotors. F_1_ synthesizes/hydrolyses ATP, and F_O_ utilizes/produces ion motive force. The torque generated by F_O_ is transmitted to F_1_ by the rotating central shaft (γεc_10_) and *vice versa*. Subunit γ is the most extended portion of the central shaft extending from the globular domain in contact with F_O_ to the top of F_1_, as evident from the pioneering [1] and the following crystal structures (e.g. [2], [3]). Although subunits γεc_10_ rotate as a whole they are elastically deformed by the torque between the two motors, and this intrinsic elastic buffer smoothes the cooperation of the two differently stepping motors (3 steps in F_1_, 10 steps in F_O_ from *Escherichia coli*) for high kinetic efficiency [4]. For recent reviews about structure and function of the F-type ATP synthase see [5]–[7].

**Figure 1:**
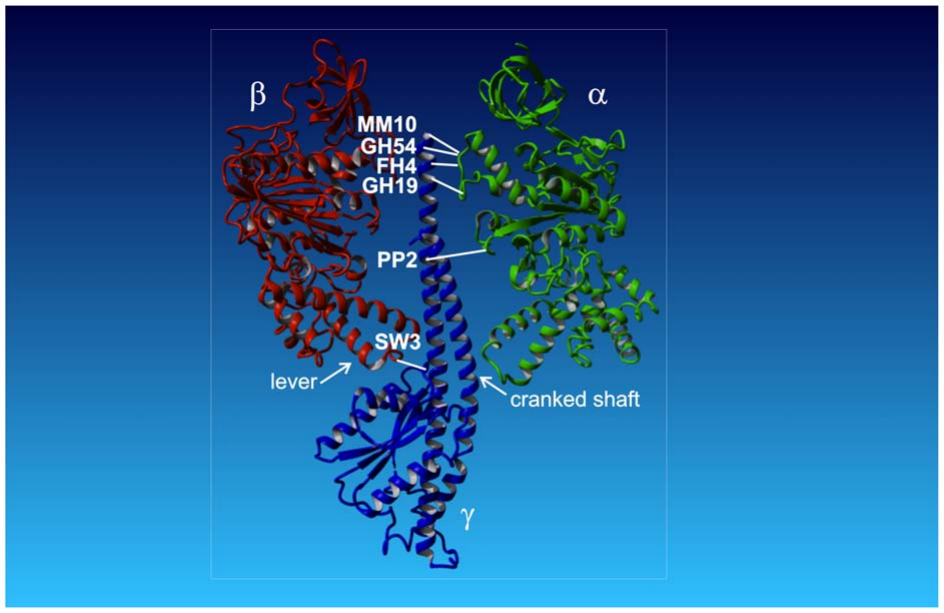
Model of *E. coli* F-ATPase. The model shows subunits α (green), β (red), and γ (blue), and the cross-link positions between subunits γ and α/β. For the sake of clarity subunit γ and only one copy each of subunit α and β are shown. Subunits δ and ε are omitted. The white lines show the positions of the cross-link sites in the respective mutants. Due to the three-fold symmetry subunit γ can form a cross-link with any of the three copies of α/β (original named E, DP, and TP [1]), the exact α/β subunit is not relevant for our experiments. The arrows indicate the positions of the lever with the DELSEED-sequence in subunit β, and the cranked shaft of the coiled coil in subunit γ. Throughout the text the C-terminus of subunit γ with cross-link position MM10 is referred as the top. The membrane embedded F_O_ with the c-ring (not shown) connect to the globular portion of subunit γ at the bottom. In between is the middle region with the coiled coil α-helices. The structural model is based on PDB ID: 3OAA [3].

Truncation experiments of subunit γ, starting from the C-terminus and ranging down into the N-terminal end within the coiled coil, have shown that the torque is generated at the interface between the lower portion of subunit γ and the conserved DELSEED-portion in subunit β [8]–[12]. It has been experimentally established that ATP hydrolysis drives the rotation of the C-terminus of subunit γ relative to the hydrophobic bearing formed by the pseudohexagon (αβ)_3_ (Fig. 1) [13]–[16]. On the other hand, an engineered cross-link between the rotor (C-terminus of subunit γ) and the stator ((αβ)_3_) of the F_1_-ATPase has neither impeded ATP hydrolysis, nor the ATP-driven rotation of the non-fixed portion of subunit γ [16], [17]. This observation has been interpreted to reveal the unfolding of the C-terminal α-helix to generate a swivel joint between neighboring residues. Moreover, in several experiments in F_1_ of various organisms the C- and N-termini of subunit γ were deleted without inactivating the ATPase activity [8]–[12]. It seems that only a small portion of subunit γ is necessary for torque generation.

Here, we extended the former work of our group and aimed to identify the domain on subunit γ, which is prone to being unfolded by the enzyme-generated torque. Six mutants of *Escherichia coli* F_1_-ATPase (EF_1_) were compared (Fig. 1). In this context the original cysteine-free (αβ)_3_γ-complex KH7 served as the ‘wild type’ enzyme. Each mutant contained two engineered cysteines for a rotor-to-stator cross-link formation, namely γA285C/αP280C (MM10), γG282C/αP280C (GH54), γI279C/αP281C (FH4), γL276C/αE284C (GH19), γL262C/αA334C (PP2), and γA87C/βD380C (SW3). In the former four mutants (MM10, GH54, FH4, GH19) the cross-link is located at the C-terminal end of subunit γ (top), while in the latter two mutants the cross-link is located in the middle of the C-terminal α-helix (PP2) and the bottom (SW3) near the globular portion of subunit γ (i.e. towards F_O_), respectively. The top portion of subunit γ consists of a single α-helix, while in the middle the C-terminal α-helix encounters its N-terminal counterpart. At the bottom subunit γ interacts with the DELSEED region of the β subunits, which serves as a lever to open its nucleotide-binding site. The activity of all mutants was monitored, both under reducing (no cross-link) and oxidizing (closed disulfide bridge between rotor and stator) conditions (Tab. 1), i.e. the rate of ATP hydrolysis in bulk solution, the cross-link yield in SDS-gels, and the rate of γ-rotation of single molecules was determined.

**Table 1:** Mutants and effects of cross-link formation.

EF_1_-mutant	Cross-link region	Cross-link position	ATPase activity/U/mg			Cross-link yield
			reduced	oxidized		
**KH7**	**-**	**-**	**140**	**-**		0%
MM10	**top**	γ285/α280	140	140	(100%)	> 98%
GH54	**top**	γ282/α280	102	78	(76%)	∼ 90%
FH4	**top**	γ279/α281	96	36	(38%)	∼ 85%
GH19	**top**	γ276/α284	34	9	(26%)	> 95%
PP2	**middle**	γ262/α334	70	<1	(<2%)	> 98%
SW3	**bottom**	γ87/β380	140	10	(7%)	∼ 90%

The table shows for the six EF_1_-mutants MM10, GH54, FH4, GH19, PP2, and SW3 the cross-link region and position, the ATP hydrolysis activity after reduction and oxidization, and the cross-link yield. By re-reducing the oxidized samples the activity could be restored (GH54: 93%, FH4: 45%, GH19: 100%). The wild type KH7 denotes the enzyme without cysteines for cross-linking the rotor to the stator. The data for KH7, MM10, PP2, and SW3 were taken from [17].

## Materials and Methods

All restriction enzymes were obtained from New England Biolabs (Frankfurt/Main, Germany) or Fermentas (St. Leon-Rot, Germany). Oligonucleotide primers were synthesized by MWG (Ebersberg, Germany). All chemicals were of the highest grade commercially available.

### Cloning

All plasmids of *E. coli* F_1_-ATP synthase were derived from pKH7 [17]–[19] (all wild type cysteines were replaced by alanines, a Histidine_6_-tag was added to the N-terminal end of subunit β, and one cysteine, γK109C, was introduced for binding an actin filament in the rotation assay). Site-directed mutagenesis was carried out using PCR. The plasmid pMM6 (αP280C, γK109C) [17] was used as a template for the generation of pFH5 (αP280C, γK109C, γG282C) using the primer 5′-CCGAGATCGTCTCGTGTGCCGCCGCGG-3′ and its complement. The primer 5′-CGAACCCGATCCGAAGTGTCTGCTGGA TACCCTGC-3′ and its complement were used to introduce an additional cysteine in subunit γ (γA213C) to improve the binding of the actin filament, resulting in the plasmid pGH54 (αP280C, γK109C, γA213C, γG282C). The plasmid pGH50 (αP281C, γK109C) was generated analogous to pMM6 using the primer 5′-CTGCTCCGTCGTCCGTGTGGACGTGAAGCATTC-3′ and its complement. The plasmid pGH50 was used as template to generate pFH4 using the primer 5′-CAGGAACTCACCGAGTGTGTCTCGGGGGCCGCCG-3′ and its complement to introduce γI279C. The template plasmids were cut using KpnI/SacI. The resulting fragment was introduced into the plasmid pBluescript II SK (Agilent Technologies, Santa Clara, USA) for mutagenesis-PCR. After verification of the introduced mutation via sequencing, the resulting plasmid was cut using KpnI/SacI, whereas the desired fragment was ligated into the corresponding template plasmid. Mutations were again verified by sequencing. The resulting plasmid was named pFH4 (αP281C, γK109C, γI279C). The plasmid pKG11 (αE284C, γK109C, γL276C) was kindly provided by K. Gumbiowski (University of Osnabrück, Germany). An additional mutation (γA213C) was introduced in pKG11 as described above, resulting in the plasmid pGH19.

### Expression and Purification

Plasmids for EF_1_ were transformed into *E. coli* DK8 cells [20] and grown overnight on minimal medium. Purification of EF_1_ was performed as described before [17]. Membranes were isolated and purified essentially according to [21]. EF_1_ was extracted by treatment with 1 mM EDTA in the presence of 0.5 mM DTT and applied to an anion-exchange column (Tosoh Fractogel TSK-DEAE 650(S), Toyo Soda, Darmstadt, Germany) equilibrated with buffer A (50 mM Tris/H_2_SO_4_, 10% (v/v) methanol, pH 7.4). EF_1_ was eluted from the column using a stepwise gradient of 0.5 M Na_2_SO_4_ in buffer A. Enzyme-containing fractions (eluted at about 150 mM Na_2_SO_4_) were combined, stabilized with 0.1 mM Mg^2+^-ATP, and reduced with 1 mM DTT. Aliquots were further purified by size exclusion chromatography, using PD-10 columns (Amersham Pharmacia Biotech), which were equilibrated with buffer B (20 mM MOPS, 5 mM MgCl_2_, 50 mM KCl, 10% (v/v) glycerol, pH 7.5). For the rotation assay an equimolar amount of biotin-PEAC5-maleimide was used for the biotinylation of mutants (20 min incubation at room temperature) at residue γC109 and γA213C. The reaction was stopped by adding 2 mM of N-acetylcysteine. Then samples were applied to a Ni-NTA affinity chromatography column (5 mg of protein/ml Ni-NTA-agarose) equilibrated with the same buffer. After washing with buffer B (containing 20 mM imidazole), pure EF_1_ was eluted with 200 mM imidazole in buffer B.

### Protein concentration

Protein concentrations were determined by using a modified assay described by Sedmak and Grossberg [22]. After addition of the reaction solution samples were incubated for 5 min at room temperature. Protein concentrations were determined photometrically at 595 nm (Hewlet-Packard, Böblingen, Germany) and compared to a reference curve of 5–40 µg/ml lysozyme.

### SDS-PAGE

SDS-PAGE was performed using the Laemmli system with a 12.5% separating gel and a non-oxidizing sample buffer. 3–5 µg protein was separated applying 200 V for 1 h in an electrophoresis chamber (Biorad, Hercules, USA). Staining was carried out with coomassie brilliant blue R-250. For reduction, oxidation, and re-reduction proteins where incubated overnight with 10 mM dithiothreitol (DTT), 400 µM Ellman's reagent (DTNB), and 30 mM DTT, respectively. The cross-link yield of scanned gels was determined using Adobe Photoshop (Adobe Systems Incorporated, San Jose, USA). The overall intensity of boxes with the same size fitted around the corresponding bands of each lane of the SDS-PAGE was determined. Background intensity was subtracted, and obtained values were compared. The percentages of gamma cross-links were determined from the changes of intensity of the γ-bands in the oxidized state in comparison to the reduced state.

### Immunoblot

For immunoblotting proteins were transferred to a PVDF-membrane after SDS-PAGE, applying 500 mA for 1 h in a blotting tank (Carl Roth, Karlsruhe, Germany). Polyclonal primary mouse and rabbit antibodies against EF_1_-α and EF_1_-γ were used at dilutions of 1∶200,000 and 1∶400,000, respectively. Peroxidase-conjugated secondary monoclonal antibodies against primary antibodies (diluted 1∶10,000) and the Lumi-LightPlus Western Blotting-Kit (Roche, Mannheim, Germany) were used for visualization by chemiluminescence. Films were developed on a Konica SRX-101A (Konica, München, Deutschland).

### ATPase hydrolysis activity

ATPase activity was determined colorimetrically as described previously [17], [23]. Proteins (10 µg/ml) in 50 mM Tris-HCl, pH 8.0, 3 mM MgCl_2_, and 10 mM sodium-ATP were incubated for 5 min at 37°C, before the reaction was stopped by the addition of trichloroacetic acid, and the released P_i_ was determined colorimetrically. The activity of reduced and oxidized samples was determined after exposure overnight at room temperature to 20 mM DTT and 200 µM DTNB, respectively. For control experiments re-reduction was achieved by incubating the oxidized samples with 20 mM DTT for 2 hours at room temperature.

### Rotation Assay and Video Microscopy

We used the same microscopic setup, and rotation assay with fluorescently labeled F-actin filaments as before [17]. Samples were filled into flow cells consisting of two coverslips (bottom: 26×76 mm^2^, top: 21×26 mm^2^, thickness: 0.15 mm, Menzel-Gläser/ProLabor, Georgsmarienhütte, Germany) separated by double adhesive tape. Protein solutions were infused in the following order (50 µl per step, 4 min incubation): 1) 0.8 µM Ni^2+^-nitrilotriacetic acid-horseradish peroxidase conjugate (Ni-NTA-HRP) in 20 mM MOPS/KOH (pH 7.0), 50 mM KCl, 5 mM MgCl_2_ (buffer A); 2) 10 mg/ml bovine serum albumin in buffer A; 3) wash with buffer A; 4) 2 µg/ml EF_1_ in buffer A; 5) wash with buffer A; 6) 0.5 µM streptavidin in buffer A; 7) wash with buffer A; 8) 200 nM biotinylated, tetramethylrhodamin-labeled short F-actin filaments in buffer A (7 min incubation); 9) wash with buffer A; and 10) 20 mM glucose, 0.2 mg/ml glucose oxidase, 50 µg/ml catalase, 5 mM ATP in buffer A (reaction buffer). Reducing conditions were achieved by adding 0.5% (v/v) 2-mercaptoethanol to the reaction buffer, which was substituted by 4–8 mM DTNB under oxidizing conditions. For re-reduction of oxidized samples 20 mM DTT was added to the reaction buffer. An inverted fluorescence microscope (IX70, Olympus, lens PlanApo x100/1.40 oil, fluorescence cube: MWIG) connected to a silicone intensified tube camera (C 2400-08, Hamamatsu Photonics Germany, Herrsching am Ammersee, Germany) and a VHS-PAL video recorder with a frame rate of 25 frames/s were used to record the rotating filaments. Recorded videos were digitalized using an A/D converter (Pinnacle Systems, München, Germany) and analyzed with a self-written software in Matlab (Math Works, Ismaning, Germany).

## Results

Six EF_1_ mutants (MM10, GH54, FH4, GH19, PP2, and SW3) each containing two opposing cysteines, one on subunit γ and the other one on either subunit α or β (Tab. 1), were analyzed for (i) their respective cross-link yield, and (ii) the ATP hydrolysis activity in bulk solution as well as on the single molecule level. The cross-link yields were determined from SDS-gels, while the hydrolysis activities were determined colorimetrically or by a videomicroscopic rotation assay, respectively. The data of the three new mutants (GH54, FH4, and GH19) were compared to the results of the mutants MM10, PP2, and SW3, and the wild type KH7 that have been published before [17].

### SDS-PAGE and immunoblots

**Figure 2:**
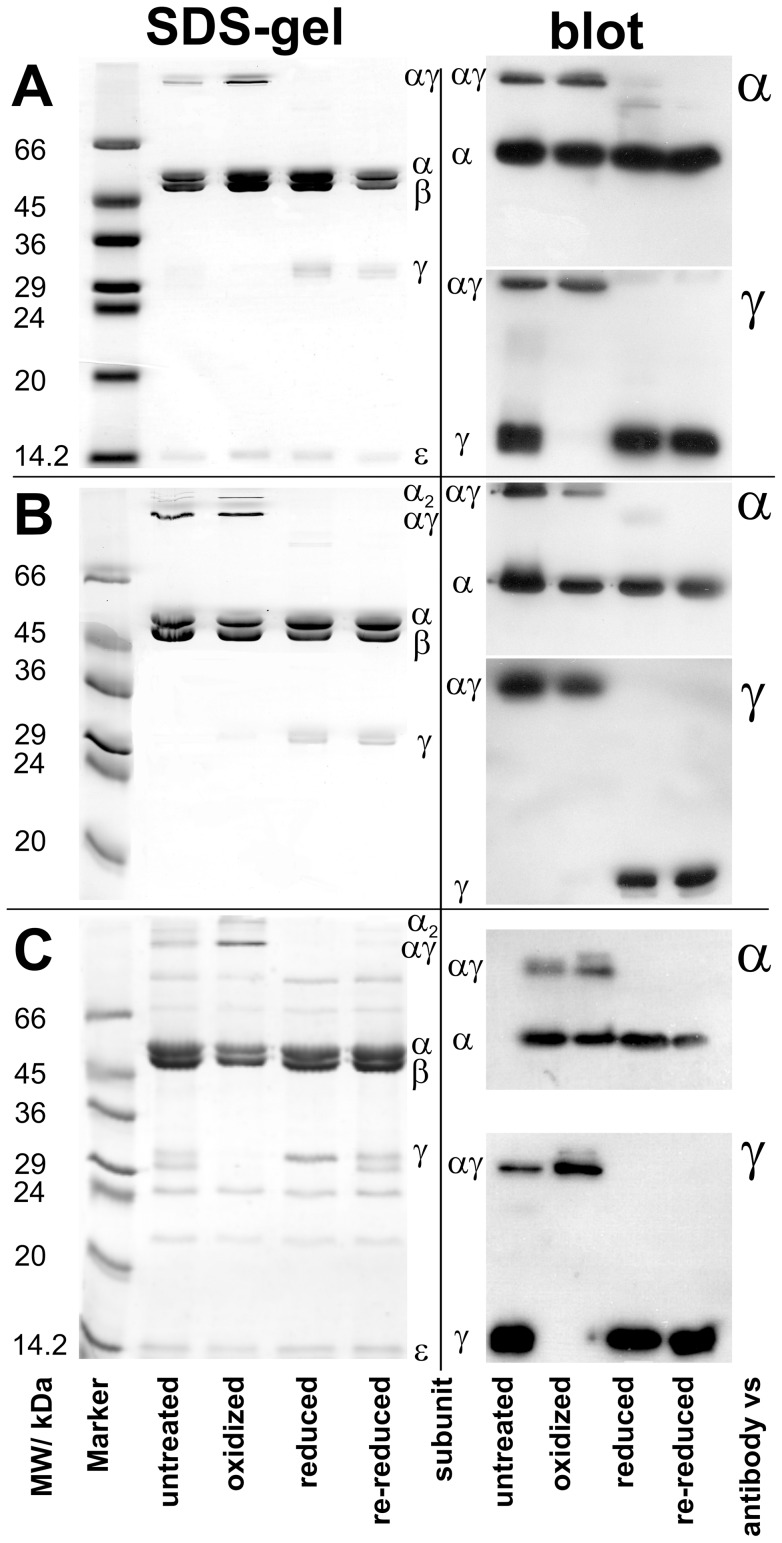
SDS-gels and immunoblots of EF_1_ mutants. Gels and Blots for GH54, FH4, and GH19 are shown in A, B, and C, respectively. For each mutant the SDS-gel is shown on the left, while the respective blots are shown on the right, on top against subunit α, and at the bottom against subunit γ. Samples are shown untreated, after oxidation with 400 µM DTNB, after reduction with 10 mM DTT, and re-reduced (after previous oxidation) with 30 mM DTT. The samples were incubated overnight with the respective chemical.

Fig. 2 demonstrates by SDS-PAGE (Fig. 2A–C, left) and immunoblot (Fig. 2A–C, right) the ability of the three new mutants GH54, FH4, and GH19 to form a disulfide bridge. (Gels and immunoblots for MM10, PP2, and SW3 were shown before [17].) Reduction and oxidation was enforced by treating the proteins overnight with 10 mM DTT and 400 µM DTNB, respectively, before applying the samples to the SDS-PAGE. The SDS gels in Fig. 2A–C (left) show the bands of the α, β, γ, and ε subunits of EF_1_ in the reduced and re-reduced (after oxidation) state. Subunit δ (and in FH4 (Fig. 2B) also subunit ε) was not visible and probably lost during protein purification. However, subunits δ and ε are not necessary for ATP hydrolysis in F_1_. In the oxidized state the band of subunit γ disappeared. Instead two new high molecular bands appeared that were attributed to the γα and α_2_ cross-link. Given the enzymes stoichiometric ratio of α to γ (3∶1) the monomeric α-band was still visible. Without added reducing or oxidizing agent (due to partial oxidation by atmospheric oxygen) all bands, monomeric and cross-linked, showed up in untreated samples, except for GH54 (Fig. 2B), where subunit γ is fully oxidized by atmospheric oxygen due to longer incubation times, and therefore not visible as a monomeric band.

Immunoblots against subunits α and γ (Fig. 2A–C, right) corroborated these results. Oxidized samples showed almost no monomeric γ-band on immunoblots against subunit γ, but an αγ-band only. As we do not know the location of the epitopes of the polyclonal antibodies we cannot rule out the possibility that some antibodies do not recognize the cross-linked subunits. In light of this, the immunoblots were not used for quantification; instead they qualitatively corroborated the quantitative result from the SDS-gels.

From the intensity changes of the γ-bands we estimated a cross-link yield for all mutants under oxidizing conditions of at least 90%, except for FH4, where it was 85% (see right column in Table 1). To check whether cross-links formed in the holoenzyme under oxidizing conditions or between single subunits in the SDS-gel, we determined the intensity of the α_2_-bands. The yield of α_2_ served as a marker for cross-links formed by the denatured enzyme in SDS as it cannot be formed in the native enzyme by monomeric α-subunits due to their distance. The yield of the most intense α_2_-band was determined to be approximately 16% of total α (i.e. monomeric α + αγ + α_2_), while the yield of monomeric α was four times larger. If cross-link formation in the SDS-gel would be the natural choice of subunits we would expect the α_2_-band to be the one of the highest intensity. Therefore, it is highly unlikely that cross-links were formed in large amounts by subunits of the denatured enzyme in the SDS-gel. Although we expected the yield of the αγ-band to be 33% of total α, because of the 3∶1 ratio of α to γ, we found only 20%. However, as no trace of a γ_2_-band was visible on any SDS-gel or immunoblot we assume that almost all γ-subunits formed a cross-link with the α subunits. Moreover, it is difficult to determine the intensity of the monomeric α-band, as the digital image might be overexposed or not well resolved from the β-band. Therefore, we account the deviations from the theoretical figures to the uncertainty of determining the α-bands intensities.

**Figure 3:**
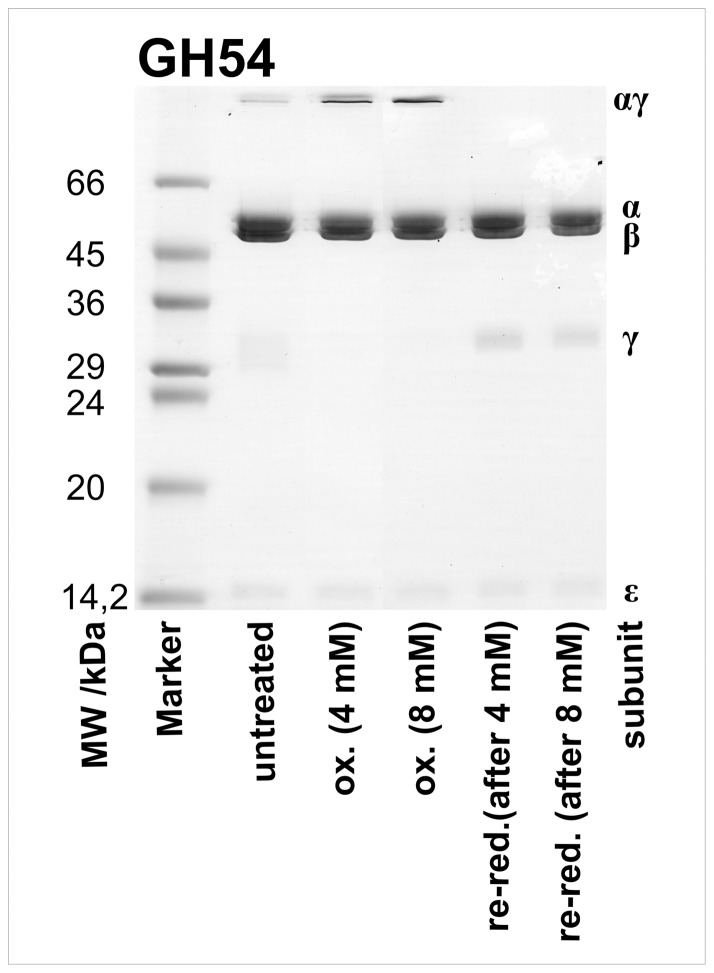
SDS-gel of the mutant GH54 under rotation assay conditions. Two samples of GH54 were oxidized (ox.) with 4 mM or 8 mM DTNB for 12 minutes, and afterwards re-reduced (re-red.) with 20 mM DTT for 12 minutes, to simulate the conditions in the rotation assay.

Oxidizing conditions in the rotation assay (see below) differed from those used for SDS-gels and bulk activity tests, i.e. the incubation time was only a few minutes instead of hours. To compensate for shorter incubation times we used higher concentrations of DTNB. To check whether these conditions affected the ability of the mutants to form cross-links we performed SDS-PAGE analysis under rotation assay conditions, i.e. oxidation and re-reduction of samples was achieved by incubation with 4–8 mM DTNB and 20 mM DTT for 12 minutes, respectively. Figure 3 shows the gel for the mutant GH54. The high molecular αγ-band appeared under oxidizing conditions, while the monomeric γ-band was only visible under reducing conditions. The lane with untreated sample showed all bands due to partial oxidation by atmospheric oxygen. This result confirmed that cross-links were formed quantitatively in the rotation assay.

### ATP hydrolysis activity in bulk solution

The bulk ATPase hydrolysis activity was discerned colorimetrically from the concentration of released phosphate. Table 1 summarizes the activities of the six double cysteine mutants, and for comparison of the wild type KH7. The data from different stocks of mutant EF_1_ were standardized by comparison of the respective activity data with those of the wild type enzyme KH7 directly after protein purification. In the reduced state the double cysteine substitution alone, without formation of a disulfide bridge, can reduce the hydrolysis rate up to fourfold. The oxidation of the engineered cysteines with concomitant formation of a disulfide bridge between rotor and stator reduces the activity further. This reduction was gradual for the first four mutants (MM10, GH54, FH4, and GH19) and practically total when the cross-link was placed in the middle (PP2) or at the bottom (SW3) of subunit γ. In addition, we checked for the reversibility of disulfide bridge formation. The activity of oxidized samples was restored after incubation for two hours with 20 M DTT.

### Rotation assay with single molecules

**Figure 4:**
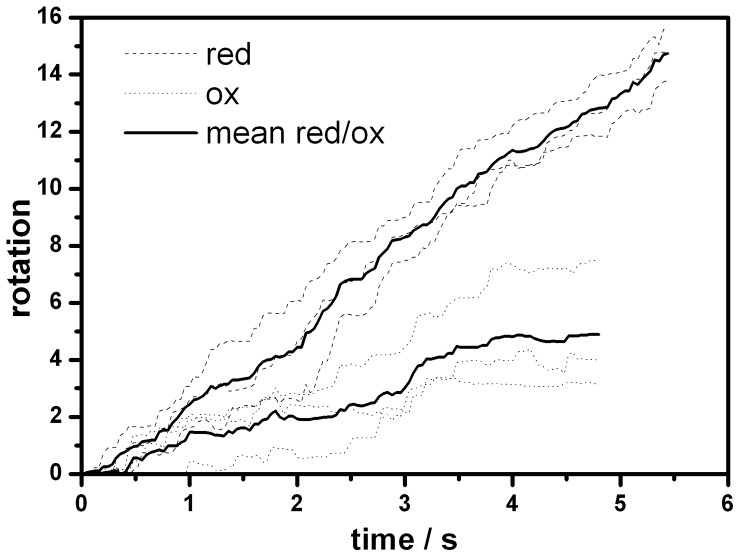
Rotary trajectories of reduced and oxidized F_1_ molecules. Trajectories of three active single molecules of GH54 driven by ATP hydrolysis both in the reduced (dashed line) and in the oxidized (dotted line) state, respectively. The mean trajectories for each of both states are shown by the solid lines.

According to the results from bulk measurements the most interesting mutants in terms of rotation of the fixed C-terminal end of subunit γ in the oxidized state were GH54 and FH4, while the cross-link activity of GH19 was the lowest of the three new mutants (Tab. 1). Therefore, we extended our research on the former two mutants by recording the rotation of single molecules of subunit γ relative to the immobilized (αβ)_3_ as previously for MM10 [17], [19]. The (αβ)_3_γ-complex was bound via Histidine_6_-tags in each β-subunit to a Ni-NTA-HRP modified cover slip. A short biotinylated, fluorescent actin filament (length 0.4–1 µm) was attached to subunit γ (γ109C, γ213C) via a cysteine-biotin-streptavidin-biotin-link to visualize its rotational movement. After addition of 5 mM ATP under reducing conditions up to 5% of the filaments started to rotate. Only these filaments were considered further. We checked whether enzymes, that stopped rotating after formation of a cross-link, were still active. We could reactivate oxidized enzymes by re-reducing the cysteines. In multiple cycles of reduction, oxidation, and re-reduction enzymes were active, inactive, and active again, respectively [24]. When changing to oxidizing conditions by addition of 4 mM DTNB (8 mM DTNB for FH4) for 10 minutes, 16 out of 29 (55%) molecules of the mutant GH54 continued to rotate while 13 stopped, typically after 2 minutes. The rotation in the still active subset could be observed significantly longer (for at least 10 minutes). The observation time was limited only by the bleaching of the fluorescent dyes under oxidizing condition. For FH4 only 3 out of 11 (27%) molecules remained active. In addition, in both cases the rotational rate decreased, typically by 60% (see Fig. 4). These data indicate that not only a fraction of the molecules is inactive, but that the activity of each active molecule is lowered, i.e. the movement of the rotor shaft is hampered.

The above single molecule activity data do not immediately relate to bulk activity data as commonly observed. Single molecule experiments are highly selective as they focus on active enzymes only, while in bulk phase measurements an unknown fraction of inactive enzymes can distort the data. Although these figures are different from those of the bulk measurement (Tab. 1) they corroborate the finding that the bulk portion of subunit γ carries out ATP driven rotation despite of a rotor-stator cross-link in the mutants MM10, GH54, and FH4.

## Discussion

We found that a cross-link between the top of the rotor (subunit γ) and the stator ((αβ)_3_) of F_1_ does not necessarily totally inhibit its ATP hydrolysis activity, but gradually reduces the rate up to fourfold (GH19), provided that the lock site on subunit γ is not farther than nine residues away from its C-terminal end. A cross-link at the penultimate residue of the C-terminal end (γ285C, MM10) was even without any effect on the activity. In contrast, a cross-link of residues γ262C (PP2, middle) or γ87C (SW3, bottom) with the stator subunits practically extinguished the hydrolysis activity of F_1_.

Three different lines of evidence support our observation. First, SDS-gels showed a cross-link yield of >85%. Second, bulk phase experiments revealed an activity of cross-linked mutants of at least 26% compared to wild type EF_1_ that could be restored after re-reducing the samples. Third, rotation assay experiments support our conclusions on a single molecule level. Not only did we find single molecules still rotating despite oxidation, but furthermore was the rotational rate reduced by 60%, indicating that rotation was impaired by the cross-link. However, as we observed only a small number of molecules the statistics are only qualitative comparable with bulk phase experiments.

What is the reason for this unusual behavior? For steric reasons alone the rotation of subunit γ around the engineered disulfide bond can be excluded because that would imply the dragging around the now obliquely oriented residues up to the C-terminal end in the before snugly fitting hydrophobic bearing. It is apparent from the crystal structure [1] (Fig. 1) that there is no space within (αβ)_3_ for such a deformation. On the other hand, at the top subunit γ is linked to a loop in subunit α that might provide some flexibility to the movement, enabling the tip of subunit γ to rotate on a ‘leash’. However, this is very unlikely for the following reason. Czub and Grubmüller [25] have shown by molecular dynamics simulation (MD) that the respective portion of subunit γ is at least four times more flexible than the opposing loop in subunit β (and therefore it is likely to be true for subunit α also because of its homology to subunit β), i.e. any torque applied would first result in a deformation of subunit γ before subunit α is affected. Disulfide bond cleavage upon ATP hydrolysis can also be excluded, because the standard dissociation energy of a single disulfide bond (∼200 kJ/mol) greatly exceeds the standard free energy of ATP hydrolysis (<60 kJ/mol) [4].

**Figure 5:**
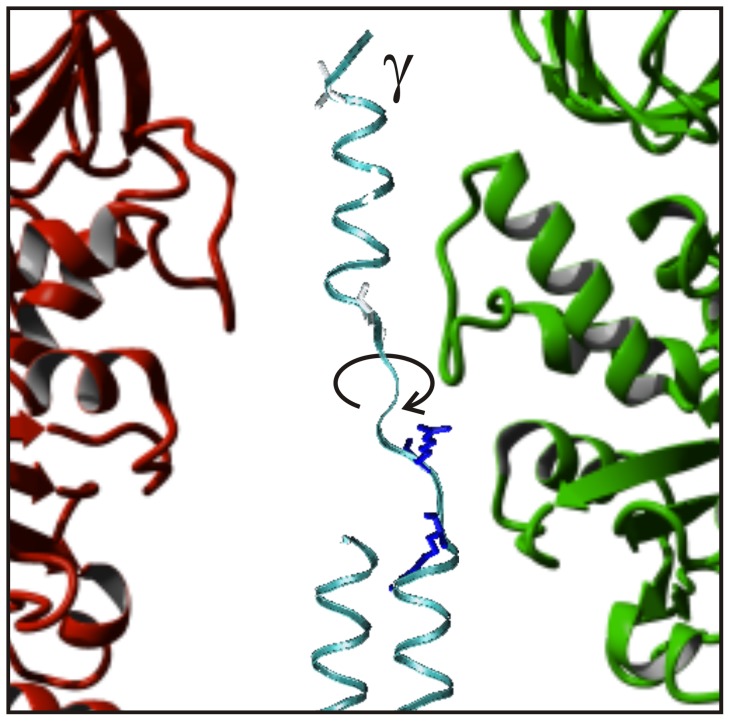
Still picture of a molecular dynamics simulation of unwinding subunit γ. The calculation by D. Cherepanov was performed as described in Gumbiowski *et al.* [17]. In short a torque of 56 pNnm was applied to the last 30 residues of subunit γ (MM10), which was fixed at residue γ285C. The calculation was done by NAMD2 [32] and CHARMM22 [33]. The picture shows the uncoiled C-terminal end of subunit γ within the same hydrophobic bearing of subunits α and β as in Fig. 1.

When the penultimate residue of the C-terminal end of subunit γ (γ285C, MM10) is locked to subunit α uncoiling of its C-terminal α-helix, as suggested previously [17], is a more reasonable explanation. Figure 5 shows a snapshot of a simulation that demonstrated the unwinding of this domain within the hydrophobic bearing of (αβ)_3_. The peptide backbone twists around the N-C_α_ and C_α_-C' bonds, the dihedral angles φ and ψ of the Ramachandran plot, respectively. The Ramachandran angles of the two C-terminal residues γG282 and γA284 are particularly susceptible to twisting motion. It was shown by molecular dynamics calculations [17] that on a nanosecond timescale the α-helix can rotate in particular around the Ramachandran angle φ of these two residues. The activation barrier for this rotation was 25–30 kJ/mol. The high torque apparently generated by ATP-hydrolyzing EF_1_ was sufficient to uncoil the C-terminal α-helix of subunit γ and to overcome the Ramachandran activation barriers. However, simulations cannot account for timescales of ms, the time domain of the active enzyme. At the two positions γ279C (FH4) and γ276C (GH19), below γ282–286 (the flexible top region of subunit γ), the cross-link impaired the ATP driven rotation. Still, some activity remained suggesting that the α-helix can be unwinded farther down by the same mechanism.

In contrast, a cross-link farther down at the middle position γ262C (PP2) inhibited the rotation totally. The inhibitory cross-link is positioned where the N-terminal α-helix meets its C-terminal counterpart (at γ268) to form an antiparallel coiled coil. This section is not prone to uncoiling, probably because the torque is not high enough to disrupt the interactions between the two α-helixes of the coiled coil.

At the bottom (γ87C, SW3) subunit γ was cross-linked to the region in β that is responsible for the opening and closing of the nucleotide-binding site. At this position the flexibility of subunit γ is needed for the regulation of the catalytic reaction (see below). Therefore, it is understandable that a cross-link at this site also totally inhibits the rotation of subunit γ.

In conclusion, subunit γ consists of three portions, namely (i) a globular portion at the bottom facing the membrane, and interacting with subunit ε, (ii) an antiparallel coiled coil in the middle, and (iii) a singular α-helix at the top C-terminal end. (i) The globular portion at the bottom, together with subunit ε [11], establishes the contact with the c-ring of F_O_. It is the elastically most compliant domain of this enzyme, and the major elastic buffer for power transmission between F_O_ and F_1_ [24]–[26]. The elastic power transmission is a prerequisite for the high kinetic efficiency of the two coupled stepping rotary motors [4], [27]–[29]. (ii) In the middle the N-terminal α-helix forms an antiparallel coiled coil with the C-terminal α-helix up to residue γ268. Several residues of the coiled coil exhibit strong non-covalent coupling with the stator during ATP hydrolysis, especially the N-terminal region of subunit γ (γ20-40) with the DELSEED-sequence in subunit β (β394–400) [11], [25]. Mechanically the DELSEED-sequence acts as a lever that is pushed by the cranked coiled coil of subunit γ (Fig. 1) to open the nucleotide-binding site at the interface of each βα-pair [1], [30], [31]. (iii) The C-terminal α-helix at the top portion, embedded in the hydrophobic bearing formed by the upper part of (αβ)_3_ [1], interacts only weakly with the stator [25] (Fig. 5). Its torsional spring constant has been determined to 750 pNnm by single-molecule fluctuation experiments [24], and 620 pNnm by MD [25]. This is rather stiff compared to the elastically most compliant domain between F_O_ and F_1_ (20 pNnm [24], [26] to 90 pNnm [25]). In the intact enzyme the C-terminus rotates with the other portions of subunit γ, as evident from experiments with a fluorescent dye coupled to the C-terminus [13], [14], and also by biochemical cross-linking techniques [16]. However, this portion can be deleted without totally impeding rotational activity. Up to 12 C-terminal residues can be deleted in EF_1_ [8], up to 20 residues in F_1_ from chloroplasts [10], and up to 43 C-terminal and 22 N-terminal residues in thermophilic F_1_ (TF_1_) [9]. Recently it was show that subunit γ of TF_1_ consisting only of the first 36 N-terminal residues can still catalyze ATP hydrolysis [11]. The C-terminal portion stabilizes the complex rather than participating in torque generation.

At first sight one might argue that the great torsional stiffness of the central stalk that we previously reported in Sielaff *et al*. [24], namely 750 pNnm, is at odds with the ready unfolding of the C-terminal α-helix that is reported in the present work and also with certain data and interpretations in references [12] and [25]. The work from our group (this work and [24]) and the one in references [12] and [25] relate to enzyme molecules from different organisms, namely from EF_1_, TF_1_, and bovine mitochondria (MF_1_), respectively. The molecules differ in the total length of subunit γ, i.e. 286 residues in EF_1_, 283 residues in TF_1_, and 272 residues in MF_1_. Sielaff *et al*. [24] observed a hard spring constant of the shaft (750 pNnm) from residue 17 (γ270) onwards to the bottom, while in the present work we observed the unwinding of the C-terminal α-helix for the top 13 residues (i.e. above γ273), and inhibition of rotation when locked at γ262 or below. There is no inconsistency between the former two, instead it seems as if the C-terminal α-helix might be compliant at the top and stiff farther down. Czub and Grubmüller [25] have simulated the experiments in ref. [24] by MD with MF_1_ over a time range of 100 ns. They found subunit γ very soft at the top 13 residues (see their Fig. 3A), and being stiff below that portion (when the SS-bridge was closed at position γ259 of MF_1_). Their simulation results are compatible with both experimental ones, in [25] and in the present work. The group of Kinosita [9], [12] has studied the ability of a C-terminal truncated subunit γ mutant TF_1_ to drive rotation and found the following: (i) The rate of rotation in mutant enzymes was diminished compared to wild type enzymes owing mainly to stumbling at *modulo* 120° positions (saturating ATP concentration). (ii) Mutants, which C-terminus was shortened by 14 residues, showed no change in torque compared to wild type (40 pNnm). (iii) In mutants that were truncated by 21 to 36 residues the torque during progression between dwells was halved compared to the wild type (20 pNnm). They have interpreted this observation by claiming that the pulling action of the (αβ)_3_-moeity on the C-terminal end of subunit γ produces about one half of the total torque, which is lost after truncation. A consequence of this interpretation is that the central shaft should be rather stiff from residue 14 onwards to the bottom, while the upper part that provide no torque can remain soft. This interpretation is consistent with the results in [24], [25], and the present work.

Previous work has shown that the rotation of the shaft in the hydrophobic bearing in the native enzyme [13], [14], [16] is the natural option for such stabilization. However, the present work shows that a swivel joint within the single α-helical domain of subunit γ is another viable option, should the former one be blocked.

## References

[1] Abrahams JP, Leslie AGW, Lutter R, Walker JE (1994) The structure of F_1_-ATPase from bovine heart mitochondria determined at 2.8 Å resolution. Nature 370: 621–628. 8065448 10.1038/370621a0

[2] Rees DM, Leslie AG, Walker JE (2009) The structure of the membrane extrinsic region of bovine ATP synthase. Proc Natl Acad Sci U S A 106: 21597–21601. 19995987 10.1073/pnas.0910365106 PMC2789756

[3] Cingolani G, Duncan TM (2011) Structure of the ATP synthase catalytic complex (F(1)) from Escherichia coli in an autoinhibited conformation. Nat Struct Mol Biol 18: 701–707. 21602818 10.1038/nsmb.2058 PMC3109198

[4] Pänke O, Cherepanov DA, Gumbiowski K, Engelbrecht S, Junge W (2001) Viscoelastic dynamics of actin filaments coupled to rotary F-ATPase: Torque profile of the enzyme. Biophys J 81: 1220–1233. 11509339 10.1016/S0006-3495(01)75780-3 PMC1301604

[5] Junge W, Sielaff H, Engelbrecht S (2009) Torque generation and elastic power transmission in the rotary F(O)F(1)-ATPase. Nature 459: 364–370. 19458712 10.1038/nature08145

[6] von Ballmoos C, Cook GM, Dimroth P (2008) Unique rotary ATP synthase and its biological diversity. Annu Rev Biophys 37: 43–64. 18573072 10.1146/annurev.biophys.37.032807.130018

[7] von Ballmoos C, Wiedenmann A, Dimroth P (2009) Essentials for ATP synthesis by F_1_F_0_ ATP synthases. Annu Rev Biochem 78: 649–672. 19489730 10.1146/annurev.biochem.78.081307.104803

[8] Müller M, Pänke O, Junge W, Engelbrecht S (2002) F_1_-ATPase: The C-terminal end of subunit γ is not required for ATP hydrolysis-driven rotation. J Biol Chem 277: 23308–23313. 11964400 10.1074/jbc.M201998200

[9] Furuike S, Hossain MD, Maki Y, Adachi K, Suzuki T, et al (2008) Axle-less F_1_-ATPase rotates in the correct direction. Science 319: 955–958. 18276891 10.1126/science.1151343

[10] Sokolov M, Lu L, Tucker W, Gao F, Gegenheimer PA, et al (1999) The 20 C-terminal amino acid residues of the chloroplast ATP synthase γ subunit are not essential for activity. J Biol Chem 274: 13824–13829. 10318787 10.1074/jbc.274.20.13824

[11] Mnatsakanyan N, Hook JA, Quisenberry L, Weber J (2009) ATP synthase with its gamma subunit reduced to the N-terminal helix can still catalyze ATP synthesis. J Biol Chem 284: 26519–26525. 19636076 10.1074/jbc.M109.030528 PMC2785340

[12] Hossain MD, Furuike S, Maki Y, Adachi K, Suzuki T, et al (2008) Neither helix in the coiled coil region of the axle of F1-ATPase plays a significant role in torque production. Biophys J 95: 4837–4844. 18708468 10.1529/biophysj.108.140061 PMC2576389

[13] Sabbert D, Engelbrecht S, Junge W (1996) Intersubunit rotation in active F-ATPase. Nature 381: 623–626. 8637601 10.1038/381623a0

[14] Sabbert D, Engelbrecht S, Junge W (1997) Functional and idling rotatory motion within F-ATPase. Proc Natl Acad Sci U S A 94: 4401–4405. 9114001 10.1073/pnas.94.9.4401 PMC20734

[15] Noji H, Yasuda R, Yoshida M, Kinosita K (1997) Direct observation of the rotation of F-ATPase. Nature 386: 299–302. 9069291 10.1038/386299a0

[16] Müller M, Gumbiowski K, Cherepanov DA, Winkler S, Junge W, et al (2004) Rotary F_1_-ATPase. Is the C-terminus of subunit gamma fixed or mobile? Eur J Biochem 271: 3914–3922. 15373837 10.1111/j.1432-1033.2004.04328.x

[17] Gumbiowski K, Cherepanov D, Müller M, Pänke O, Promto P, et al (2001) F-ATPase: forced full rotation of the rotor despite covalent cross-link with the stator. J Biol Chem 276: 42287–42292. 11533065 10.1074/jbc.M106884200

[18] Kuo PH, Ketchum CJ, Nakamoto RK (1998) Stability and functionality of cysteine-less F1F0 ATP synthase from *Escherichia coli* . FEBS Lett 426: 217–220. 9599011 10.1016/s0014-5793(98)00337-8

[19] Noji H, Häsler K, Junge W, Kinosita K Jr, Yoshida M, et al (1999) Rotation of Escherichia coli F(1)-ATPase. Biochem Biophys Res Comm 260: 597–599. 10403811 10.1006/bbrc.1999.0885

[20] Klionsky DJ, Brusilow WSA, Simoni RD (1984) In vivo evidence for the role of the epsilon subunit as an inhibitor of the proton-translocating ATPase of *Escherichia coli* . J Bacteriol 160: 1055–1060. 6238948 10.1128/jb.160.3.1055-1060.1984 PMC215818

[21] Wise JG (1990) Site-directed mutagenesis of the conserved beta subunit tyrosine 331 of *Escherichia coli* ATP synthase yields catalytically active enzymes. J Biol Chem 265: 10403–10409. 2141332

[22] Sedmak JJ, Grossberg SE (1977) A rapid, sensitive, and versatile assay for protein using Coomassie brilliant blue G250. Anal Biochem 79: 544–552. 68686 10.1016/0003-2697(77)90428-6

[23] LeBel D, Poirier GG, Beaudoin AR (1978) A convenient method for the ATPase assay. Anal Biochem 85: 86–89. 147037 10.1016/0003-2697(78)90277-4

[24] Sielaff H, Rennekamp H, Wächter A, Xie H, Hilbers F, et al (2008) Domain compliance and elastic power transmission in rotary F(O)F(1)-ATPase. Proc Natl Acad Sci U S A 105: 17760–17765. 19001275 10.1073/pnas.0807683105 PMC2584700

[25] Czub J, Grubmüller H (2011) Torsional elasticity and energetics of F1-ATPase. Proc Natl Acad Sci U S A 108: 7408–7413. 21502534 10.1073/pnas.1018686108 PMC3088579

[26] Wächter A, Bi Y, Dunn SD, Cain BD, Sielaff H, et al (2011) Two rotary motors in F-ATP synthase are elastically coupled by a flexible rotor and a stiff stator stalk. Proc Natl Acad Sci U S A 108: 3924–3929. 21368147 10.1073/pnas.1011581108 PMC3053995

[27] Cherepanov DA, Mulkidjanian A, Junge W (1999) Transient accumulation of elastic energy in proton translocating ATP synthase. FEBS Lett 449: 1–6. 10225416 10.1016/s0014-5793(99)00386-5

[28] Pänke O, Rumberg B (1999) Kinetic modeling of rotary CF_0_F_1_-ATP synthase: storage of elastic energy during energy transduction. Biochim Biophys Acta 1412: 118–128. 10393255 10.1016/s0005-2728(99)00059-6

[29] Junge W, Pänke O, Cherepanov D, Gumbiowski K, Müller M, et al (2001) Inter-subunit rotation and elastic power transmission in F_o_F_1_-ATPase. FEBS Lett 504: 152–160. 11532447 10.1016/s0014-5793(01)02745-4

[30] Menz RI, Walker JE, Leslie AG (2001) Structure of bovine mitochondrial F(1)-ATPase with nucleotide bound to all three catalytic sites: implications for the mechanism of rotary catalysis. Cell 106: 331–341. 11509182 10.1016/s0092-8674(01)00452-4

[31] Masaike T, Koyama-Horibe F, Oiwa K, Yoshida M, Nishizaka T (2008) Cooperative three-step motions in catalytic subunits of F(1)-ATPase correlate with 80 degrees and 40 degrees substep rotations. Nat Struct Mol Biol 15: 1326–1333. 19011636 10.1038/nsmb.1510

[32] Kale L, Skeel R, Bhandarkar M, Brunner R, Gursoy A, et al (1999) NAMD2: Greater scalability for parallel molecular dynamics. Journal of Computational Physics 151: 283–312.

[33] Brooks BR, Bruccolori RE, Olafson BD, States DJ, Swaminathan S, et al (1983) CHARMM: A Program for Macromolecular Energy, Minimization, and Dynamics Calculations. J Comput Chem 4: 187–217.

